# Supplementary material for: Association of an increase in serum albumin levels with positive 1-year outcomes in acute decompensated heart failure: A cohort study
Source: PLoS One. 2020 Dec 28;15(12):e0243818. doi: 10.1371/journal.pone.0243818 (PMC7769473; doi:10.1371/journal.pone.0243818)
Supplement: S1 File — (DOCX) [file pone.0243818.s002.docx]

**S1 File**

**Ethical approval of other participating centers**

Shiga General Hospital (approval number: 20141120-01), Tenri Hospital (approval number: 640), Kobe City Medical Center General Hospital (approval number: 14094), Hyogo Prefectural Amagasaki General Medical Center (approval number: Rinri 26-32), National Hospital Organization Kyoto Medical Center (approval number: 14-080), Mitsubishi Kyoto Hospital (approved 11/12/2014), Okamoto Memorial Hospital (approval number: 201503), Japanese Red Cross Otsu Hospital (approval number: 318), Hikone Municipal Hospital (approval number: 26-17), Japanese Red Cross Osaka Hospital (approval number: 392), Shimabara Hospital (approval number: E2311), Kishiwada City Hospital (approval number: 12), Kansai Electric Power Hospital (approval number: 26-59), Shizuoka General Hospital (approval number: Rin14-11-47), Kurashiki Central Hospital (approval number: 1719), Kokura Memorial Hospital (approval number: 14111202), Kitano Hospital (approval number: P14-11-012), and Japanese Red Cross Wakayama Medical Center (approval number: 328).
