# Supplementary material for: Association of an increase in serum albumin levels with positive 1-year outcomes in acute decompensated heart failure: A cohort study
Source: PLoS One. 2020 Dec 28;15(12):e0243818. doi: 10.1371/journal.pone.0243818 (PMC7769473; doi:10.1371/journal.pone.0243818)

**S1 Fig.** **Adjusted survival curves.**

(A) A composite of all cause death and hospitalization for heart failure. (B) All-cause death. (C) Heart failure hospitalization. We adjusted outcomes for the same variables in COX hazard model.


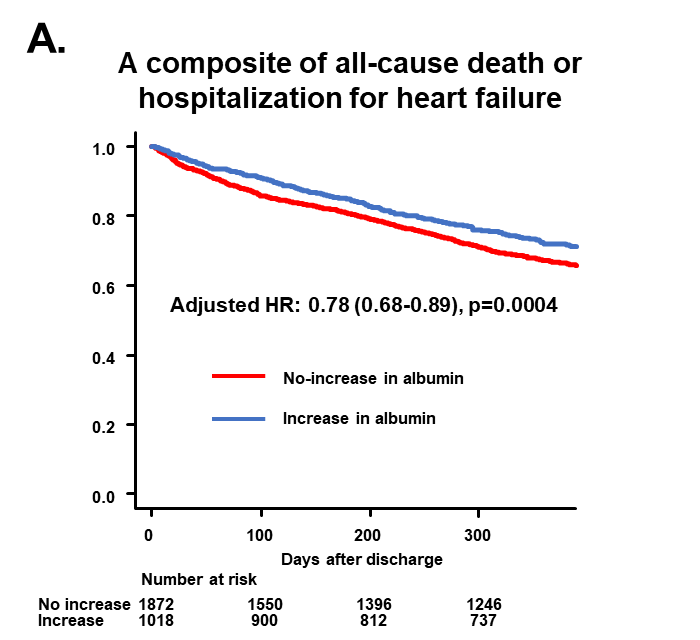


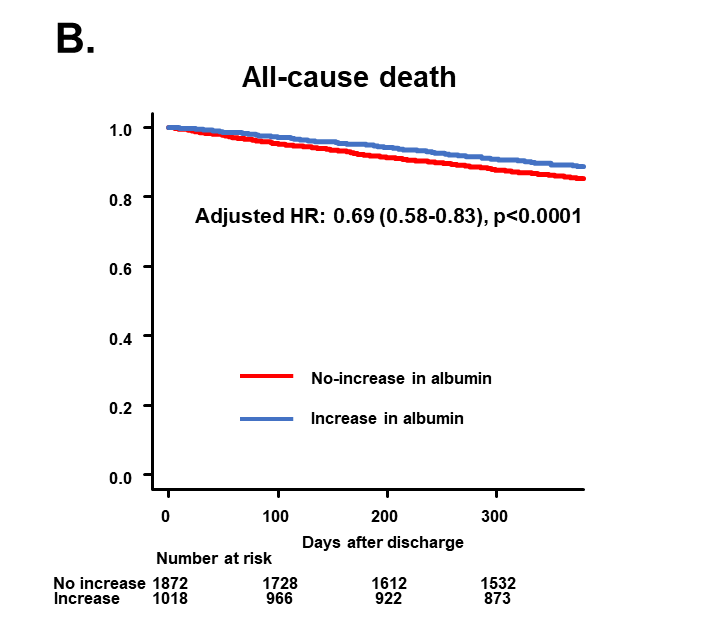


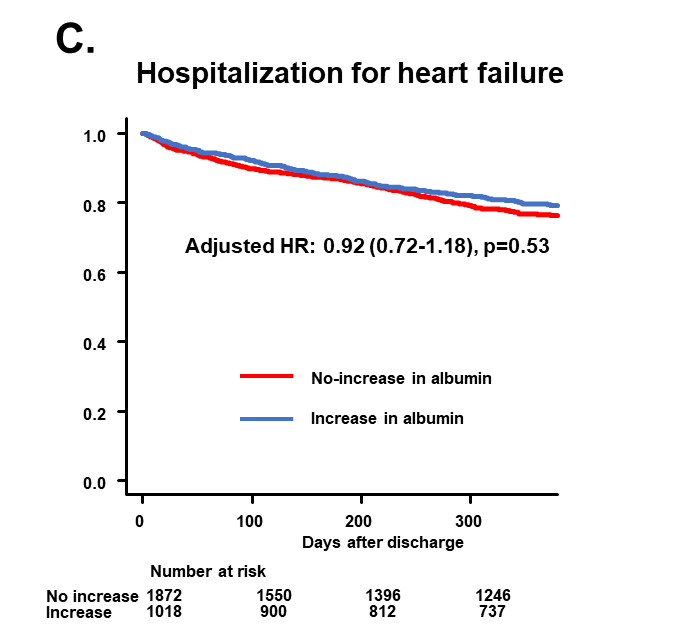

Supplement: S1 Fig — (A) A composite of all cause death and hospitalization for heart failure. (B) All-cause death. (C) Heart failure hospitalization. We adjusted outcomes for the same variables in COX hazard model. (DOCX) [file pone.0243818.s003.docx]
