# Supplementary material for: Association of an increase in serum albumin levels with positive 1-year outcomes in acute decompensated heart failure: A cohort study
Source: PLoS One. 2020 Dec 28;15(12):e0243818. doi: 10.1371/journal.pone.0243818 (PMC7769473; doi:10.1371/journal.pone.0243818)

**S2 Fig.** **Kaplan Meier curves for the primary outcome measure stratified by quartiles of the percent change of albumin levels.**


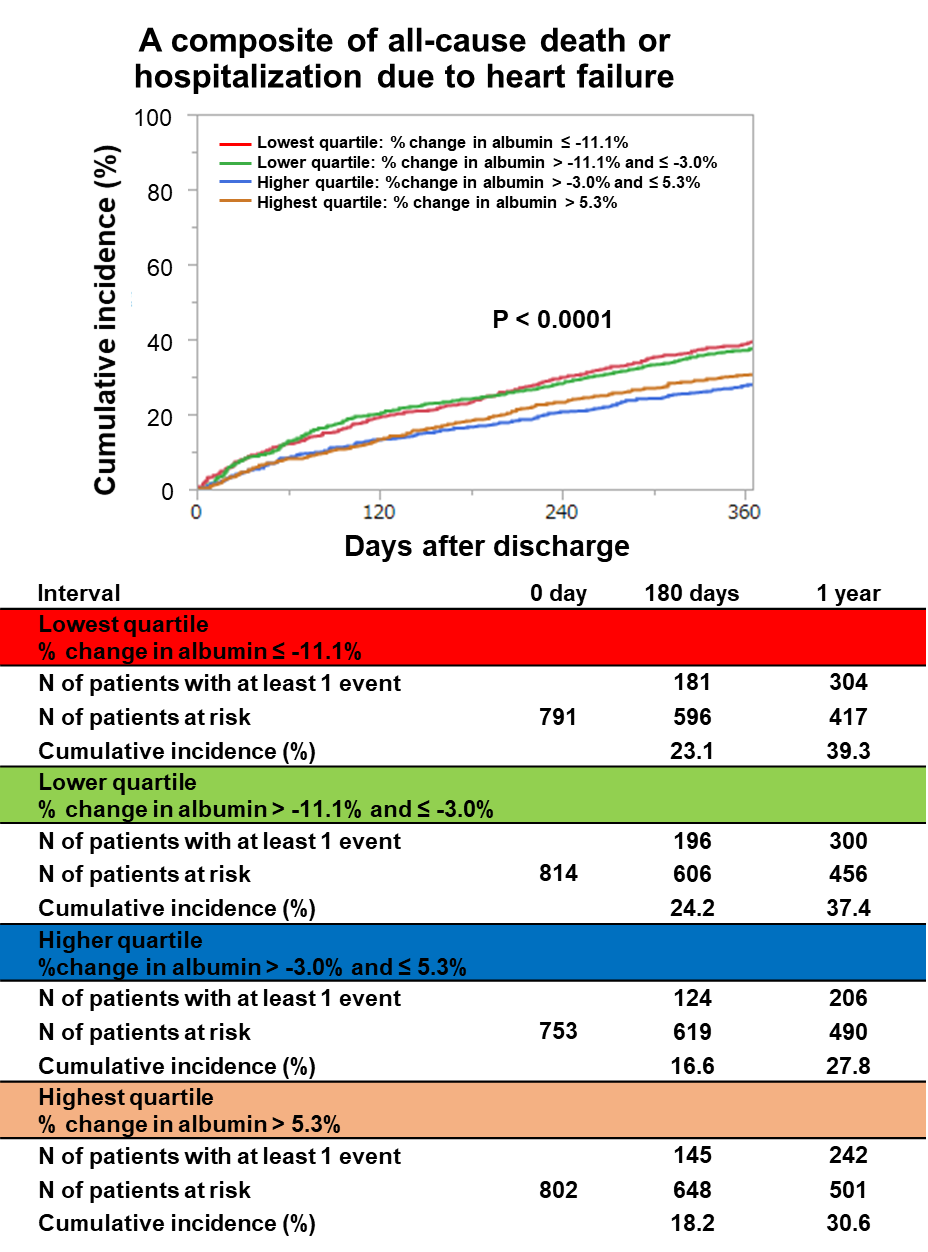

Supplement: S2 Fig — (DOCX) [file pone.0243818.s004.docx]
