# Supplementary material for: Association of an increase in serum albumin levels with positive 1-year outcomes in acute decompensated heart failure: A cohort study
Source: PLoS One. 2020 Dec 28;15(12):e0243818. doi: 10.1371/journal.pone.0243818 (PMC7769473; doi:10.1371/journal.pone.0243818)
Supplement: S1 Table — (DOCX) [file pone.0243818.s005.docx]

**S1 Table. Baseline characteristics of the patients with versus without albumin data**

| **Variables** | **Available albumin data**  **(N=3160)** | **Unavailable albumin data**  **(N= 568)** | **P value** |
| --- | --- | --- | --- |
| Age | 80 [72-86] | 80 [71-86] | 0.4919 |
| Men | 1748 (55.3) | 309 (54.4) | 0.7140 |
| BMI < 22 kg/m^2^ | 1398 (46.5) | 241 (44.2) | 0.3504 |
| ACS | 173 (5.5) | 33 (5.8) | 0.7646 |
| Prior HF | 1120 (35.9) | 201 (37.6) | 0.4650 |
| Hypertension | 2311 (73.1) | 388 (68.3) | 0.0191 |
| Diabetes | 1196 (37.9) | 201 (35.4) | 0.2790 |
| COPD | 427 (13.5) | 62 (10.9) | 0.1049 |
| Prior MI | 726 (23.0) | 112 (19.7) | 0.0907 |
| Prior stroke | 512 (16.2) | 81 (14.3) | 0.2622 |
| Malignancy | 461 (14.6) | 76 (13.4) | 0.4758 |
| Current smoking | 380 (12.2) | 73 (13.1) | 0.5766 |
| Systolic BP < 100mmHg | 185 (5.9) | 44 (7.8) | 0.0882 |
| HR < 60 bpm | 215 (6.8) | 37 (6.6) | 0.9275 |
| AF at presentation | 1318 (41.7) | 238 (41.9) | 0.9631 |
| eGFR<30mL/min/1.73m^2^ | 845 (26.7) | 139 (24.7) | 0.3501 |
| Na < 135 mEq/L | 381 (12.1) | 53 (9.5) | 0.0863 |
| Anemia | 2114 (67.0) | 348 (61.7) | 0.0157 |
| LVEF < 40% | 1158 (36.7) | 225 (39.9) | 0.1561 |

BMI = body mass index; ACS = acute coronary syndrome, HF = heart failure; COPD = chronic obstructive pulmonary disease; MI = myocardial infarction; BP = blood pressure; HR = heart rate; AF = atrial fibrillation/flutter; eGFR = estimated glomerular filtration rate; LVEF = left ventricular ejection fraction.
