# Supplementary material for: Association of an increase in serum albumin levels with positive 1-year outcomes in acute decompensated heart failure: A cohort study
Source: PLoS One. 2020 Dec 28;15(12):e0243818. doi: 10.1371/journal.pone.0243818 (PMC7769473; doi:10.1371/journal.pone.0243818)
Supplement: S2 Table — (DOCX) [file pone.0243818.s006.docx]

**S2 Table. Patient characteristics based on the quartiles of the percent change of albumin levels**

| Variables | Lowest quartile  (≤ -11.1%)  (N=791, 25.0%%) | Lower quartile  (> -11.1% and ≤ -3.0%)  (N=814, 25.8%) | Higher quartile  (> -3.0% and ≤ 5.3%)  (N=753, 23.8%) | Highest quartile  (> 5.3%)  (N=802, 25.4%) | P value | Cochran-Armitage trend test | N of patients analyzed |
| --- | --- | --- | --- | --- | --- | --- | --- |
| Clinical characteristics |  |  |  |  |  |  |  |
| Age >80 years* | 466 (58.9) | 455 (55.9) | 383 (50.9) | 345 (43.0) | <0.0001 | <0.0001 | 3,160 |
| Men* | 424 (53.6) | 444 (54.6) | 418 (55.5) | 462 (57.6) | 0.41 | 0.10 | 3,160 |
| BMI <22 kg/m^2^* | 382 (52.0) | 366 (47.2) | 305 (42.2) | 345 (44.5) | 0.0014 | 0.0009 | 3,009 |
| Prior hospitalization for heart failure* | 271 (34.7) | 290 (36.2) | 276 (37.0) | 283 (35.7) | 0.82 | 0.62 | 3,124 |
| Etiology |  |  |  |  |  |  |  |
| Dilated cardiomyopathy | 53 (6.7) | 77 (9.5) | 91 (12.1) | 119 (14.8) | <0.0001 | <0.0001 | 3,160 |
| Acute coronary syndrome* | 63 (8.0) | 38 (4.7) | 29 (3.9) | 43 (5.4) | 0.003 | 0.02 | 3,160 |
| Medical history |  |  |  |  |  |  |  |
| Atrial fibrillation or flutter* | 310 (39.2) | 347 (42.6) | 337 (44.8) | 324 (40.4) | 0.12 | 0.48 | 3,160 |
| Hypertension* | 608 (76.9) | 619 (76.0) | 540 (71.7) | 544 (67.8) | <0.0001 | <0.0001 | 3,160 |
| Diabetes mellitus* | 303 (38.3) | 317 (38.9) | 258 (34.3) | 318 (39.7) | 0.13 | 0.96 | 3,160 |
| Prior myocardial infarction* | 196 (24.8) | 178 (21.9) | 173 (23.0) | 179 (22.3) | 0.53 | 0.35 | 3,160 |
| Prior stroke* | 121 (15.3) | 142 (17.4) | 116 (15.4) | 133 (16.6) | 0.61 | 0.75 | 3,160 |
| Current smoking* | 81 (10.5) | 80 (10.0) | 100 (13.5) | 119 (15.0) | 0.006 | 0.001 | 3,104 |
| Chronic lung disease* | 118 (14.9) | 122 (15.0) | 91 (12.1) | 96 (12.0) | 0.12 | 0.03 | 3,160 |
| Liver cirrhosis* | 12 (1.5) | 10 (1.2) | 9 (1.2) | 9 (1.1) | 0.90 | 0.49 | 3,160 |
| Malignancy* | 124 (15.7) | 130 (16.0) | 110 (14.6) | 97 (12.1) | 0.11 | 0.03 | 3,160 |
| Living alone* | 149 (18.8) | 168 (20.6) | 169 (22.4) | 180 (22.4) | 0.24 | 0.05 | 3,160 |
| Ambulatory* | 606 (77.5) | 635 (78.7) | 606 (81.0) | 641 (80.6) | 0.27 | 0.07 | 3,132 |
| Systolic blood pressure <90 mmHg* | 23 (2.9) | 13 (1.6) | 13 (1.7) | 29 (3.6) | 0.03 | 0.34 | 3,153 |
| Heart rate <60 bpm* | 59 (7.5) | 49 (6.1) | 47 (6.3) | 60 (7.5) | 0.53 | 0.94 | 3,142 |
| HFrEF (LVEF <40%)* | 293(37.2) | 288 (35.5) | 290 (38.5) | 287 (35.8) | 0.59 | 0.88 | 3,153 |
| eGFR <30 mL/min/1.73m^2^* | 247 (31.2) | 233 (28.6) | 191 (25.4) | 174 (21.7) | 0.0001 | <0.0001 | 3,160 |
| Sodium <135 mEq/L* | 116 (14.7) | 91 (11.2) | 76 (10.1) | 98 (12.2) | 0.04 | 0.11 | 3,155 |
| Anemia* | 497 (62.8) | 550 (67.7) | 514 (68.4) | 553 (69.0) | 0.04 | 0.01 | 3,157 |
| Albumin, g/dl * |  |  |  |  |  |  |  |
| Q1: ≤ 3.2 g/dl | 140 (17.7) | 222 (27.3) | 194 (25.8) | 431 (53.7) | <0.0001 | <0.0001 | 3,160 |
| Q2: > 3.2 g/dl and ≤ 3.5 g/dl | 174 (22.0) | 152 (18.7) | 197 (26.2) | 190 (23.7) | 0.004 | 0.06 | 3,160 |
| Q3: ≥3.5 g/dl and ≤ 3.8 g/dl | 200 (25.3) | 200 (24.6) | 202 (26.8) | 146 (18.2) | 0.0003 | 0.004 | 3,160 |
| Q4: > 3.8 g/dl | 277 (35.0) | 240 (29.5) | 160 (21.3) | 35 (4.4) | <0.0001 | <0.0001 | 3,160 |
| Medications at discharge |  |  |  |  |  |  |  |
| ACE-I or ARB* | 362 (45.8) | 385 (47.3) | 347 (46.1) | 342 (42.6) | 0.28 | 0.17 | 3,160 |
| Beta blocker* | 277 (35.0) | 335 (41.2) | 314 (41.7) | 310 (38.7) | 0.03 | 0.14 | 3,160 |
| MRA | 123 (15.6) | 133 (16.3) | 149 (19.8) | 139 (17.3) | 0.14 | 0.14 | 3,160 |
| Hospital stay >16 days* | 433 (54.7) | 351 (43.1) | 341 (45.3) | 411 (51.3) | <0.0001 | 0.32 | 3,160 |

*Risk-adjusting variables selected for COX hazard model including albumin quartiles, which are identical to Table 1.
