# Supplementary material for: Association of an increase in serum albumin levels with positive 1-year outcomes in acute decompensated heart failure: A cohort study
Source: PLoS One. 2020 Dec 28;15(12):e0243818. doi: 10.1371/journal.pone.0243818 (PMC7769473; doi:10.1371/journal.pone.0243818)
Supplement: S3 Table — (DOCX) [file pone.0243818.s007.docx]

**S3 Table. Outcomes based on the quartiles of the percent change of albumin levels**

|  | N of patients with  event/N of patients at  risk (Cumulative  1-year incidence  [%]) | Unadjusted | | Adjusted | |
| --- | --- | --- | --- | --- | --- |
|  |  | HR (95% CI) | P value | HR (95% CI) | P value |
| A composite of　all-cause death or hospitalization due to heart failure |  |  |  |  |  |
| Lowest quartile | 360/791  (39.3%) | 1.0  (reference) |  | 1.0  (reference) |  |
| Lower quartile | 359/814  (37.4%) | 0.95  (0.82-1.09) | 0.45 | 0.97  (0.83-1.15) | 0.78 |
| Higher quartile | 263/753  (27.8%) | 0.68  (0.58-0.80) | <0.0001 | 0.70  (0.59-0.83) | <0.0001 |
| Highest quartile | 287/802  (30.6%) | 0.71  (0.61-0.83) | <0.0001 | 0.72  (0.60-0.86) | 0.0004 |
| All-cause death |  |  |  |  |  |
| Lowest quartile | 220/791  (21.6%) | 1.0  (reference) |  | 1.0  (reference) |  |
| Lower quartile | 204/814  (18.4%) | 0.85  (0.71-1.03) | 0.10 | 0.87  (0.71-1.08) | 0.21 |
| Higher quartile | 147/753  (14.8%) | 0.64  (0.52-0.79) | <0.0001 | 0.70  (0.56-0.88) | 0.003 |
| Highest quartile | 153/802  (14.4%) | 0.61  (0.50-0.75) | <0.0001 | 0.59  (0.47-0.75) | <0.0001 |
| Hospitalization due to heart failure |  |  |  |  |  |
| Lowest quartile | 211/791  (24.6%) | 1.0  (reference) |  | 1.0  (reference) |  |
| Lower quartile | 231/814  (25.4%) | 1.04  (0.87-1.26) | 0.66 | 1.06  (0.87-1.29) | 0.56 |
| Higher quartile | 167/753  (18.2%) | 0.75  (0.61-0.91) | 0.005 | 0.75  (0.60-0.93) | 0.01 |
| Highest quartile | 183/802  (20.1%) | 0.78  (0.64-0.95) | 0.013 | 0.82  (0.65-1.03) | 0.09 |

HF=heart failure, CI=confidence interval, HR=hazard ratio.

The patients were stratified into quartiles of the percent change of albumin levels: the lowest quartile (≤ -11.1%), the lower quartile (> -11.1% and ≤ -3.0%), the higher quartile (> -3.0% and ≤ 5.3%), and the highest quartile (> 5.3%).
