# Supplementary material for: Association of an increase in serum albumin levels with positive 1-year outcomes in acute decompensated heart failure: A cohort study
Source: PLoS One. 2020 Dec 28;15(12):e0243818. doi: 10.1371/journal.pone.0243818 (PMC7769473; doi:10.1371/journal.pone.0243818)
Supplement: S4 Table — (DOCX) [file pone.0243818.s008.docx]

**S4 Table. Subgroup analysis with or without acute coronary syndrome**

|  | | Increase in albumin | No-increase in albumin | Unadjusted | | Adjusted | | |
| --- | --- | --- | --- | --- | --- | --- | --- | --- |
|  |  | N of patients with events/N of patients at  risk (Cumulative 1-year incidence) | N of patients with events/N of patients at  risk (Cumulative 1-year incidence) | HR (95% CI) | P value | HR (95% CI) | P value | P value for interaction |
| A composite of death or HF hospitalization | ACS | 10/53  (13.5%) | 40/120  (22.0%) | 0.49  (0.23-0.94) | 0.03 | 0.25  (0.09-0.63) | 0.002 | 0.08 |
|  | No ACS | 372/1030  (30.4%) | 847/1957  (37.0%) | 0.78  (0.69-0.88) | <0.0001 | 0.80  (0.70-0.92) | 0.001 |  |
| All-cause death | ACS | 4/53  (4.0%) | 25/120  (12.7%) | 0.32  (0.09-0.82) | 0.01 | 0.13  (0.03-0.61) | 0.01 | 0.03 |
|  | No ACS | 200/1030  (14.9%) | 495/1957  (19.2%) | 0.73  (0.61-0.85) | <0.0001 | 0.72  (0.60-0.87) | 0.0005 |  |
